# Supplementary material for: Are carbon emissions trading and green financial instruments synergistic? -Comprehensive quantitative research based on content analysis
Source: PLoS One. 2024 Mar 7;19(3):e0298601. doi: 10.1371/journal.pone.0298601 (PMC10919601; doi:10.1371/journal.pone.0298601)
Supplement: S1 Data — (ZIP) [file pone.0298601.s001.zip › Data/地级市Z_F工作报告.zip!/╦─┤¿/├╝╔╜╩╨/└┤╫╘╣1⁄2╓┌║┼í╛┬φ┐╦╩2╛▌═°í┐_2014.docx]

各位代表：

我代表市人民政府，向大会报告工作，请予审议，并请政协委员提出宝贵意见。

2013年工作回顾

2013年，是极不寻常、极不平凡的一年。面对国际形势复杂多变、国内经济增速放缓、重大自然灾害叠加的严峻考验，我们在省委、省政府和市委的坚强领导下，在市人大、市政协的监督支持下，砥砺奋进，攻坚破难，真抓实干，较好地完成了市三届人大四次会议确定的目标任务。

——最大限度释放了发展潜能。预计（下同），全市地区生产总值（GDP）860亿元，增长11.3%；全社会固定资产投资760亿元，增长26.5%；规模以上工业增加值增长11%；社会消费品零售总额增长14%；民营经济增加值增长13.3%；民间投资占全社会投资比重达到78%。

——最大限度提升了质量效益。三产增加值增长12.4%，三次产业结构调整为16.4：57.3：26.3。地方公共财政收入63.6亿元，增长30.3%，增速居全省第2位，总量在全省升2位；东坡区、仁寿县、彭山县地方公共财政收入突破10亿元大关；广义城镇化率提高3.3个百分点；超额完成省下达的节能减排任务。

——最大限度增进了民生福祉。城镇居民人均可支配收入21782元，增长10.2%；农村居民人均纯收入9323元，增长13.2%，实现了“九连快”；新增城镇就业3.64万人；6.5万人实现脱贫；人口自然增长率3.34‰。

一年来，我们坚持“稳中求进”，紧紧围绕“三四五翻番”和“两个率先”奋斗目标，保持定力、深处着力、有序有力，大力实施“七大会战、三大工程”，全市经济社会发展保持了“稳中有进、积极向好”的良好势头。

突出转型升级，产业发展更加优化。坚持工业强市、产业兴市，着力调结构、转方式，扩大增量、盘活存量，产业发展正逐步迈向高端化、集群化、集约化。产业结构不断优化。优势产业放量增长，机械装备、精细化工、泡菜等5个产业产值突破百亿元。战略性新兴产业实现产值170亿元，在工业经济中的比重达到17%。龙头企业规模壮大，新增规模以上企业72户、限额以上商贸单位236户、50亿元企业集团1户。现代服务业发展迅速，市场消费日益活跃，全年旅游收入142.1亿元，增长28.9%。粮食总产实现“十连增”。产业质态不断优化。工业提质增效，规模以上工业实现利润58亿元，增长28%；入库税金24.2亿元，增长8%。单位GDP能耗下降3.5%，万元工业增加值能耗下降8%。质量强市成效明显，品牌竞争力增强，建成中国泡菜产业技术研究院和国家级泡菜质检分中心，新增中国驰名商标1件、地理标志证明商标2件、国家地理标志产品2个，创建省级旅游度假区1个。主要农作物综合机械化水平达到56%，建成国家和省级农业标准化示范项目8个。产业布局不断优化。园区成为经济发展的主战场，聚集效应显著提升。彭山经开区、甘眉园区、视高开发区晋级百亿园区，彭山青龙物流园区成为全省唯一的省级现代物流聚集区。现代农业“两城三园七片”强力推进，岷江现代农业示范园区成为国家农业产业化示范基地，眉山奶业获全省“科教兴川示范基地”称号。

突出项目带动，发展活力更加强劲。坚持项目总抓手，投资主驱动，以大开放促大发展，经济增长的内生动力和持续活力不断增强。项目投资强力支撑。项目会战、项目竣工高潮迭起，全年集中开工重点项目182个，竣工项目137个，完成投资275亿元。100个省市重点项目，累计投资303亿元，完成目标任务的150%。全市金融机构存款余额突破1000亿元，新增信贷首次突破100亿元，成功发行了我市首支城投债券16亿元。重点区域强力突破。天府新区强力推进，新落户项目30个，招商引资到位资金95亿元。眉山现代工业新城完成固定资产投资132亿元，总部经济区建设迈出实质性步伐，彭山经开区创建国家级经开区、眉山经开区扩区调位工作全面启动。岷东新区建设全面启动，完成固定资产投资12.09亿元、招商引资到位资金12.1亿元。东坡岛整体开发有序推进，东坡湖综合整治进展良好。开放合作强力推进。央企入眉、民企入眉行动深入开展，成眉、甘眉合作亮点纷呈，全市招商引资到位资金601.2亿元，实际利用外资2亿美元，分别保持全省第2、3位。新引进IBM、华润万家、苏宁云商等世界500强、中国500强或投资10亿元以上项目24个。对外贸易创历史最好水平，进出口总额2.8亿美元，增长43%。

突出统筹城乡，发展基础更加牢固。坚持以城带乡、城乡一体，强力推进城乡要素保障、平等交换和公共资源均衡配置，依法用地，可持续发展载体进一步夯实。基础建设有力推进。“两宋荣光”眉州大道全面完成“两桥一站”和西段核心段建设，“四箭齐发”完成投资22亿元。乐雅高速眉山段建成通车。成都经济区环线、仁沐新高速眉山段成功招商，遂资眉高速眉山段加快推进。龚家堰水库扩容工程等重大水利基础设施加快建设。成德绵眉乐城际铁路眉山段、岷江汉阳航电工程建设进展顺利。城乡建设有序推进。创新“四种模式”，加快“四个转变”，推进人的城镇化，全年农村居民转变为城镇居民15.62万人，转变为产业工人8.9万人，转变为三产经营者和从业人员3.57万人，转变为现代农业业主1661户。城镇规模扩张、品质提升，新增城镇建成区面积16.17平方公里。城乡规划大会战基本实现规划一步到位。产村相融推进新村建设，建成新农村综合体11个。生态建设有效推进。围绕全域生态化、全域景区化，加快“绿海明珠、千湖之城”建设，城市饮用水水源地达标率100%，全市森林覆盖率达45%。国家园林城市、国家卫生城市和省级文明城市创建成果丰硕。

突出增进福祉，社会环境更加和谐。坚持民之所系、责之所在，政府新增财力主要用于改善民生，大力促进社会公平正义，不断提升人民群众的幸福指数。民生工程全面完成。民生支出占地方公共财政支出66%。超额完成十大民生工程，完成投资47.59亿元，超年度计划15.1个百分点。保障性住房开工5788套，开工率连续三年全省第一。养老服务体系建设全省领先，农村五保集中供养率保持全省第一。各项事业全面发展。成功创建国家技术创新工程四川省试点示范市。素质教育成效明显，义务教育巩固率达94%，高考上线率居全省前列，新引进高校总数全省第一。深入推进文化立市，成功举办市第三届运动会、第四届东坡文化节暨第五届四川泡菜展销会，东坡宋城建设全面开工，社区书屋在全国率先实现全覆盖。医药卫生体制改革稳步推进，新农合参合率达99.93%全省第二，县级公立医院全部取消药品加成。社会保障全面加强，城乡低保应保尽保，企业退休人员基本养老金人均每月提高155.32元，城镇职工、城镇居民基本医疗保险最高支付限额分别提高到20万元/年、12万元/年。社会治理全面加强。依法治市深入推进，首批进入全国法治城市创建活动先进行列。广泛开展“双拥共建”活动，国防后备力量建设取得新成效。创新社会治理，深化平安眉山建设，继续完善社会稳定风险评估制度和社会矛盾化解“大调解”体系，创新建立维稳跟着项目走、群众来访当天办理制度，全市进京到省非正常上访连续6年保持全省末位。眉山被评为全国社会管理综合治理优秀市，“诉非衔接”改革试点工作获中期评估全国第一。圆满完成村（居）委会换届工作。全面落实 “一岗双责”，关闭小煤矿3处，煤矿企业兼并重组有序推进，全市安全生产形势持续稳定好转。

突出务实高效，自身建设更加有为。坚持依法行政、高效勤政、廉洁从政，全面推进政府治理创新，“五型”政府建设不断取得新成果。治理能力进一步增强。沉着应对自然灾害多发、突发事件频发的严峻形势，成功抵御了“4·20”芦山强烈地震、“7·9”特大暴雨洪灾。认真编制“4·20”芦山强烈地震灾后恢复重建规划，抗震救灾和灾后恢复重建取得阶段性胜利。行政效能进一步提升。深入推进行政审批制度改革，进一步简政放权，下放和取消行政审批51项。创新建立“三大服务中心”，全面完成行政权力依法规范公开运行平台建设，权力运行更加透明。规范执法自由裁量权，强化行政执法监督，政府公信力全面提高。认真办理人大代表建议、政协委员提案，满意和基本满意率达100%。廉政建设进一步加强。在全省率先建成统一的电子交易平台，政府采购引入网上竞价，公共资源交易更加规范。认真贯彻落实中央“八项规定”、省委“十项规定”和市委“六个坚持”，市级“三公”经费下降10.5%。

各位代表！

过去的一年，我们在极其艰难、极为复杂的严峻形势下，爬坡上坎、加力加劲、倍道兼程，努力开创了眉山跨越发展新局面！取得的成绩来之不易，这是省委、省政府和市委坚强领导的结果，是市人大和市政协监督支持的结果，是全市人民齐心协力、奋力拼搏的结果。在此，我谨代表市人民政府，向历任老领导、老同志，向全市人民、解放军驻眉部队和武警、消防官兵、政法干警，向关心、支持和参与眉山建设发展的社会各界朋友，表示衷心的感谢并致以崇高的敬意！

回顾一年来的进取之路，我们深深地认识到：问题不容忽视，忧患才能兴市。虽然宏观形势严峻复杂、经济整体下行、市场需求不足，是导致去年一些目标没有完成的客观原因，但是经济总量不大、运行质量不高、产业结构不优，仍是眉山跨越发展的根本问题；重大产业项目缺乏、自主创新能力不强、资源环境约束加大，仍是眉山加快发展的重要瓶颈；二元结构特征明显、城乡发展不平衡、公共服务水平偏低，仍是眉山永续发展的突出矛盾；政府职能转变不够到位、治理结构不够合理，体制机制不够完善，仍是建设现代政府的主要差距。对上述问题，我们将高度重视，认真解决。

回顾一年来的砥砺之路，我们深深地感受到：这一年，是我们有效应对各种严峻挑战，在砥砺中前行的一年。这一年，是我们追逐富裕美好和谐“眉山梦”，在探索中奋进的一年。这一年，是我们围绕实现“两个率先”目标，在跨越中突破的一年。实践证明，任何困难都压不倒眉山人民！只要我们提振精气神，凝聚正能量，以落地有声、有令必行的锐气抓执行，以逢山开路、遇河架桥的豪气抓推进，以踏石留印、抓铁有痕的勇气抓落实，就一定能够过了一山再登一峰，跨过一沟再越一壑，在眉山跨越发展的康庄大道上越走越宽广！

回顾一年来的奋斗之路，我们深深地体会到：必须坚持发展为先，任何时候都不动摇。发展是解决所有问题的关键。必须牢牢把握加快发展的主旋律，紧紧扭住第一要务不放松。只有这样，才能积跬步至千里、汇万江成大海，开创富裕美好和谐眉山新局面。必须坚持创新实干，任何时候都不懈怠。创新产生动力，实干催生希望。面对各种困难和考验，任何时候都要保持奋发有为的精神状态，勇于打开解放思想的“总闸门”，不断寻求开启成功的“金钥匙”。只有这样，才能扫清前进道路上的障碍，冲破制约发展的困境，实现眉山跨越发展新提升。必须坚持民生至上，任何时候都不忘记。民生连着民心，民心凝聚民力。必须始终把群众对美好生活的向往作为政府工作的目标。只有这样，才能让发展成果更多地惠及广大群众，赢得人民的信赖与支持，迸发出无往而不胜的磅礴力量！

2014年重点工作

2014年是深入贯彻落实党的十八届三中全会精神的开局之年，是实现 “三四五翻番”目标、全面建成小康社会的关键之年。做好今年政府工作，意义重大，影响深远。我们要牢牢把握科学发展主题、转变发展方式主线，紧紧围绕“改革创新、转型升级、提质增效”不动摇，扎实做好“改革创新突破年、产业转型升级年、统筹城乡攻坚年、产业集群招商年、创业就业惠民年”“五个主题年”工作，努力实现经济社会健康持续发展，奋力走在全省加快发展前列。

全市经济社会发展的主要预期目标为：地区生产总值增长10%；全社会固定资产投资增长17%；规模以上工业增加值增长11%；社会消费品零售总额增长13%；地方公共财政收入增长15%；城镇居民人均可支配收入增长10%；农村居民人均纯收入增长11%；城镇化率提高2.5个百分点；全面完成省下达安全生产和节能减排任务。

实现上述目标，要突出抓好以下30项重点工作。

一、致力改革开放，全面推进加快发展

改革因问题而产生，开放更是倒逼改革。只有坚持改革开放，以改革统领发展全局，把改革开放贯穿于经济社会发展各个领域各个环节，才能充分激发发展的活力和动力。

1．深化经济体制改革。实行统一的市场准入制度，建立“负面清单”管理模式，最大限度地缩小审批、核准范围，改善和优化融资结构，提高直接融资比重。积极发展各类地方性金融机构和新兴金融组织，加快推进农村信用社改革，组建县级农村商业银行。完善政府债务风险防控机制，进一步调整优化财政支出结构，改革预算制度，深入推进“营改增”试点。

2．推进统筹城乡改革。加快推进“四种模式”、“四个转变”，全域全面放开城市和建制镇落户限制。解决城镇稳定就业常住人口的落户问题，力争实现城镇常住人口基本公共服务全覆盖。全面开展农村各类产权确权，探索增加农民财产性收入途径，加快构建多种形式的新型农业经营体系。

3．充分激发民营经济活力。坚持权利平等、机会平等、规则平等，让一切创造社会财富的源泉充分涌流。大力发展混合所有制经济，鼓励民间资本平等进入金融、能源及公益性企业和公共服务等领域，支持社会资本通过特许经营等方式参与城镇基础设施建设和运营。全年民营经济增加值增长11%，继续保持全省前列。

4．全力招强引优。深入实施“376”招商战略，创新承接重大产业转移方式，围绕产业链、供应链、价值链招强引优，大力引进一批产业关联性大、带动性强的大企业、大项目。积极开展校（院）市合作、央企入眉、民企入眉等重大招商活动，力争全年新引进世界500强、中国500强或投资10亿元以上项目20个以上，招商引资到位资金632亿元、实际利用外资2亿美元以上。

5．构建开放型经济体系。加快对外开放步伐，主动融入长江经济带、丝绸之路经济带、21世纪海上丝绸之路，大力加强与长三角、珠三角、环渤海、港澳台等区域的经济合作。培育开放新优势，深入实施融入成都同城发展战略，进一步扩大成眉甘眉合作范围，加快推进成眉工业集中发展区、成眉石化园区、甘眉园区建设，力争成为次级会展中心城市。深入开展企业“走出去”战略，积极引导企业参与“万企出国门”、“川货全国行”、“惠民购物全川行动”三大活动，确保进出口贸易总额增长6%。

二、致力创新驱动，全面推进转型发展

转型是时代的主题，升级是发展的主线。只有大力实施创新驱动，换挡不失速，创新又创造，才能构建眉山现代产业体系，打造眉山经济升级版。

6．大力增强科技创新能力。加快实施企业创新主体培育工程，新增国家高新技术企业5家、省级以上创新型企业5家、省级以上企业技术中心1个，基本建成以企业为主体、以市场为导向、产学研用紧密结合的技术创新体系。完善科技成果转化平台和机制，实施重大科技成果转化项目20个，带动实现产值60亿元。深入实施人才强市战略，完善创新人才评价、使用、激励机制，加快培育和引进各类创新人才和团队。

7．打造特色产业集群。紧紧围绕七大主导产业，突出链条延伸、“两化”融合、集聚发展，深入推进百亿产业、百亿园区、百亿企业“三百工程”。大力培育和发展战略性新兴产业，实现产值210亿元，占工业比重达到19%。加快培育大企业大集团，推动企业建立现代企业制度，力争金象赛瑞、千禾味业2家企业成功上市，新增规模以上企业30户、10亿元以上企业5户，新培育省级以上品牌30个。

8．加快建设现代工业新城。加快总部经济区建设，打造核心区和配套区。加快推进阳光坚端铝业、中科兴业聚芳硫醚砜等10个重大项目建设，新开工亿元以上项目20个。完成固定资产投资150亿元，实现工业增加值增长12%以上。加快推进眉山经开区、彭山经开区创建国家级经开区。

9．积极化解产能过剩。完善激励和约束政策，加快推进陶瓷、水泥、造纸等行业整合重组，努力化解过剩产能，坚决淘汰落后产能。以高新技术和先进适用技术改造提升传统产业，推动产业转型升级，全年完成技改投入242亿元。

10．大力发展现代农业。努力提高粮食综合生产能力，切实保障粮食安全。充分依托“两城三园七片”，加快发展都市近郊型农业。大力发展特色效益农业，扎实推进现代畜牧业深化试点，办好泡菜展销会，力争全年泡菜产业产值突破120亿元，新增无公害农产品、绿色食品和有机食品品牌20个，畜禽良种化率达到97%。加强农田水利基本建设，新增灌溉面积5.58万亩，新建高标准农田10万亩。加快培育新型农业经营主体，让农民成为体面的职业，新增市级以上重点龙头企业4户、现代农业业主1500户、家庭农场50个、标准化合作社30个。

11．提升发展现代服务业。坚持传统服务业与现代服务业发展并重，大力发展电子商务、信息消费、健康服务、文化创意等新兴服务，努力实现现代服务业发展提速、比重提升、水平提高。加快推进彭山青龙、仁寿文林物流园区、IBM西南云计算中心和西南网贸港、苏宁集团西南运营基地、亚马逊电子商务运营中心等项目建设，限额以上商贸单位达到500户以上。发展壮大现代金融业，新增金融机构2家、贷款115亿元以上。积极培育新的消费热点和亮点，充分释放消费基础作用的新动能，力争创建全国信息消费试点市。促进房地产业持续健康有序发展。

12．大力发展旅游经济。整体推进东坡宋城、瓦屋山、柳江古镇、黑龙滩、三岔湖仁寿景区、彭祖山、中岩寺等重点景区开发建设，加快大峨眉旅游西环线、老峨山旅游快速通道等重点旅游项目建设，完成旅游项目投入60亿元。强力推进旅游标准化建设，启动七里坪创建国家级旅游度假区，全年实现旅游收入160亿元。

三、致力统筹城乡，全面推进一体发展

城乡二元结构是发展的最大瓶颈，城市二元结构是发展的最大障碍。只有坚持统筹城乡、一体发展，加快推进以人为核心的新型城镇化，才能形成以城带乡、城乡一体的新型城乡关系。

13．优化城镇发展体系。坚持“三规衔接、三规并举、三城一体”，完善市域新型城镇化发展规划。进一步优化城镇形态，规范城镇空间秩序，走出一条形态适宜、产城融合、城乡一体、集约高效的新型城镇化路子，成功申报一批省级试点镇。

14．完善城乡基础建设。大力构建方便快捷的综合交通网，全力打好高速公路、干线公路、轨道交通、水路通道建设攻坚战，加速推进“四箭齐发”、成都经济区环线、成德绵眉乐城际铁路、遂资眉高速、仁沐新高速、岷江航运航电等重点项目建设，开工建设连汪燕铁路，确保完成综合交通项目投资95亿元，力争100亿元。完成农村公路改善工程200公里，行政村油路水泥路通畅率达85%。加快天然气管网、电力配套设施等能源基础设施建设，大力推进“三网融合”，建设“数字眉山”，新建4G移动基站776个。

15．加快新区建设。强力推进天府新区建设，加快联合利华（四川）全球生产基地、成都信息工程学院天府校区等重大项目建设，完成固定资产投资100亿元，尽快建成宜业宜商宜居的国际化现代新区。强力推进岷东新区建设，基本形成城市道路框架，加快推进四川师范大学成都学院、四川城市职业学院等重大项目建设，完成固定资产投资13亿元。启动中心城区南北郊区建设，强力推进东坡岛整体开发，全面完成东坡湖污染综合整治工程。

16．夯实底部基础。做大做强县域经济，按照现代城市标准建设5个县城，着力推进县城新区建设。加快重点小城镇建设，抓好“百镇建设试点行动”，努力形成一批具有历史记忆、地域特色、独具风格的现代城镇和精品小镇。坚持“产村相融”推进3个省级新农村示范县和7个市级新农村示范片建设。全面启动9个幸福美丽新村建设试点，新开工建设新农村综合体20个，力争建成13个。

17．提升城乡公共服务水平。大力完善城镇功能，加快推进四大主题公园建设，完成 “两宋荣光”眉州大道改造、一环路改造等项目建设，争取启动中心城区绕城路建设。提升城市管理水平，确保成功创建国家园林城市、省级文明城市。推进城乡基本公共服务均等化和社会建设一体化，加快建设文明和谐的新型农村社区。

四、致力生态文明，全面推进绿色发展

保护生态环境就是保护生产力，改善生态环境就是发展生产力。只有执行最严格的生态红线保护制度，深入推进全域生态化，才能把绿水青山留给眉山人民，让眉山人民望得见山、看得见水、记得住乡愁。

18．狠抓环境治理。深入开展城乡环境综合治理，实施环境质量提升工程，建成环境优美示范县1个、示范乡镇4个、示范村庄45个。严格危险化学品和危险废物管理，推进造纸、食品加工行业深度治理。切实加强饮用水水源保护，加大通惠河污染综合整治力度，积极加快垃圾收集处理、生活污水处理设施建设。深入开展大气污染综合防治，可吸入颗粒物浓度下降2.5%。

19．深入推进生态建设。大力推进“绿海明珠”建设，完成交通干道多色谱林业景观带和24个景观节点建设。加强天然林资源保护，巩固退耕还林成果，森林覆盖率保持45%以上。着力加快“千湖之城”和“四湖”建设，实施引水入城、入镇、入村、入园，恢复和新增水域面积21.14平方公里。加快推进洪雅、丹棱、青神创建国家级生态县，东坡、彭山创建省级生态县。

20．进一步加强节能减排。强化环境影响评价，严格落实总量减排目标责任制。加快化工、建材等重点能耗企业节能技术改造，大力推进建筑、公共机构等领域节能降耗。

21. 努力提高土地利用效率。执行最严格的耕地保护制度和节约集约用地制度，严守耕地保护红线。全面建立产业园区土地利用绩效评估制度，探索建立项目用地全程管理机制。积极开展闲置土地和低效用地清理，盘活存量土地。

五、致力民生改善，全面推进共享发展

共享是最大的公平，民生是政府全部工作的出发点和落脚点。只有坚持严守 “底线民生”，改善 “重点民生”，保障 “基本民生”，实施 “十大民生工程”，突出办好为民实事，才能让发展成果更多更公平惠及全市人民。

22．大力促进创业就业。深入推进创业就业优先战略，全力做好高校毕业生、城镇困难人员、“9+3”藏区学生等重点群体就业工作，城镇新增就业2.6万人，城镇登记失业率控制在4.4%以内。完善全民创业服务体系，加大政策支持力度，积极创建省级创业型示范城市。

23．提高社会保障水平。继续抓好社会保险征缴扩面，实现养老、工伤、生育、医疗保险市级统筹，推进异地就医结算，实现社会保障“一卡通”。继续抓好住房公积金归集扩面，加强贷后管理，保障资金安全。加快征地拆迁安置住房建设，确保安置1.5万人。大力实施保障性住房建设，加大危旧房、城中村、棚户区改造力度，实施“百万安居工程建设行动”，全市开工建设公租房4010套、竣工1813套，棚户区危旧房改造启动5455套、完成1787套。完善多元养老服务体系，加快发展养老服务产业。健全覆盖城乡的社会救助体系。

24．推进教育事业协调发展。深化教育领域综合改革，统筹城乡义务教育资源均衡配置，深入实施素质教育，大力促进教育公平。建设现代职业教育体系，增强职业教育服务地方经济发展能力。全力抓好“学校安全三年行动”计划，新建、改扩建校舍5万平方米。广泛开展全民健身活动，大力发展群众体育。

25．加快医疗卫生事业发展。深化医药卫生体制改革，完善新农合制度和基本药物制度，探索公立医院改革试点。加强医疗卫生服务体系和能力建设，启动市人民医院二期工程建设，推进市中医院整体迁建，实现每个县有二甲及以上综合医院。加大重大传染病和地方病的防治力度，促进基本公共卫生服务均等化。

26．推进文化大发展大繁荣。深入实施“文化立市”战略，积极构建现代公共文化服务体系，广泛开展群众性文化创建活动。加强公共文化设施建设，开工建设市博物馆、文化馆和图书馆。加快文化产业发展，深入推进东坡宋城、中国竹编艺术城建设和三苏祠灾后恢复重建。繁荣艺术创作生产，加强文化遗产保护。

27．加快灾后恢复重建。强力推进“4·20”芦山强烈地震灾后恢复重建，提升发展灾区基础设施、生态建设和特色产业，推动灾区面貌和发展水平大幅提升。

28．推动社会事业全面发展。做好“十二五”规划中期评估工作和全国第三次经济普查工作，积极抓好妇女儿童、人口计生、关心下一代、民族宗教、外侨台、广播电视、气象、地方志、保密、档案、慈善、红十字会、残疾人、扶贫移民等各项社会事业。

29．切实维护公共安全。加强国防动员和后备力量建设，争创“双拥模范城”。加强食品药品安全监督管理，推进建立食品生产销售全程追溯制度，确保“舌尖上的安全”。坚持“三零”工作法抓好安全生产，坚决遏制重特大安全事故发生。大力提升防灾减灾能力，健全重大自然灾害、突发公共安全事件应急体系和处理机制。深入开展道路交通安全综合整治，深化平安眉山建设，严厉打击各类违法犯罪活动。

30．加强社会治理。大力推进法治眉山建设，强化依法治市“八大体系”建设，深入开展“六五”普法，积极创建全国法治城市。完善社会稳定风险评估、“大调解”等机制，加强群众和信访工作，维护司法公正，保障群众合法权益。

努力建设现代政府

打铁还需自身硬。全面深化改革、加快转型发展，必须大力推进政府治理能力现代化，加快建设现代政府。

建设科学理政能力。坚持用改革的精神、思路、办法来改善政府治理，寓改革创新于治理之中。创新治理方式，加快从微观管理、正面清单、行政手段为主向宏观调控、负面清单、综合手段为主转变。进一步转变发展观念，建立更加科学的指标体系和考核标准。

建设依法行政能力。深入开展法治政府创建，充分运用法治思维和法治方式做好政府工作，提高依法行政水平。严格行政决策程序，健全规范性文件、重大决策合法性审查机制。深化行政执法体制改革，落实行政执法责任制和过错责任追究制，完善行政执法程序，规范执法自由裁量权。做好行政复议、行政调解和仲裁工作。

建设高效勤政能力。加快政府职能转变，进一步简政放权，使市场在资源配置中起决定性作用和更好发挥政府作用。深化行政体制改革，完成市县区政府部门机构改革，全面推进事业单位分类改革。深化行政审批制度改革，进一步取消、下放行政审批项目。完善电子政务、中介服务平台建设，健全政务服务五级体系。进一步精简会议文件。

建设民主治政能力。严格执行人大决议决定，自觉接受人大法律监督、工作监督和政协民主监督，健全人大代表建议批评意见、政协委员提案“绿色通道”。深入推进行政协商，广泛听取各方意见，高度重视群众监督和舆论监督。完善政府信息公开和政务公开机制。

建设廉洁从政能力。坚持把权力关进制度的笼子，让权力在阳光下运行。健全反腐倡廉制度体系，坚持用制度管权管事管人。推行政府权力清单制度，依法公开权力运行流程，构建决策科学、执行坚决、监督有力的权力运行体系。加强节约型机关建设，严格落实《党政机关厉行节约反对浪费条例》、《党政机关国内公务接待管理规定》。扎实开展党的群众路线教育实践活动，坚决整治“四风”，努力实现政府清廉、干部清正。

各位代表！没有比人更高的山，没有比脚更长的路。让我们高举中国特色社会主义伟大旗帜，在省委、省政府和市委的坚强领导下，凝心聚力、开拓创新，对标先进、扎实苦干，为建设富裕美好和谐眉山而努力奋斗！
